# Prevalence of Type 2 Diabetes Mellitus among People Attending Medical Camp in a Community Hospital

**DOI:** 10.31729/jnma.4953

**Published:** 2020-05

**Authors:** Anu Kushwaha, Anuj Raj Kadel

**Affiliations:** 1 Department of Emergency Medicine and General Practice, Kathmandu Medical College Teaching Hospital, Sinamangal, Kathmandu, Nepal; 2 Kathmandu Medical College Teaching Hospital, Duwakot, Bhaktapur, Nepal

**Keywords:** *diabetes mellitus*, *prevalence*, *screening*

## Abstract

**Introduction:**

Diabetes is a health problem on the rise in developing countries like Nepal. Often in the suburban and rural areas, patients are diagnosed in the late stages with complications. The aim of this study is to find out the prevalence of diabetes type 2 in a community hospital of Nepal.

**Methods:**

This is a descriptive cross-sectional study done in a community hospital from January to March of 2019 after ethical clearance (Registration number: 150320192) from the institutional review committee of Kathmandu Medical College. Convenient sampling technique was used. Glucometer using glucose sticks is used to measure random blood sugar level and relevant questions were asked in a short interview. The data were analyzed using the Statistical Package for the Social Sciences 20 version.

**Results:**

Out of a total of 114 people, the prevalence of type 2 diabetes mellitus was 5 (4.38%). Among those 5 (4.385%) people with type 2 diabetes mellitus, 2 (1.75%) were female and 3 (2.63%) were male. The minimum age of the patient was 17 years and the maximum age was 92 years. Five out of 95 patients with mild physical activity had random blood sugar more than 200 mg/dl and five out of 46 alcoholic patients had random blood sugar levels more than 200 mg/dl. Only 1 out of 26 smokers had a random blood sugar level of more than 200 mg/dl.

**Conclusions:**

Prevalence of diabetes mellitus type 2 in our study population is quite high. Early detection of diabetes mellitus type 2 can be a good screening tool for early treatment and prevention of complications.

## INTRODUCTION

The reason behind a global rise in the incidence of type 2 diabetes mellitus may be due to higher average life expectancy, increasing trend of a sedentary lifestyle, or an overall lack of physical exercise. WHO states that global prevalence is about 9% of the adult population in 2014.^1,2^ More than 80% diabetic-related death occurs in low and middle-income countries.^3^ The international diabetic federation estimates that one-fifth of all diabetic in the world live in South East Asia. WHO projected that diabetes will the seventh leading cause of death in the world in 2013^4^ and not surprisingly we dread that it will keep rising in rank.

Currently, DM screening is infrequent in rural areas thus increasing the burden of undetected cases. Early intervention of these cases could decrease diabetes-related complications.

The aim of this study is to determine the prevalence of type 2 diabetes mellitus among the people attending medical camp in a community hospital.

## METHODS

This was the descriptive cross-sectional study conducted in a community hospital from January 2019 to March 2019 after receiving ethical approval from the Institutional Review Committee (Registration number: 150320192) of Kathmandu Medical College. The patients with age above 14 years who come for random blood sugar level testing in a medical health camp and gave consent for the study were studied. Patients with a history of diabetes mellitus, children below 14 years of age, and pregnant women are excluded from the study. Glucometer with glucose sticks was used to measure the random blood sugar level. After explaining the purpose, importance, and procedure in detail a written informed consent was obtained from all the participants. Demographic data like weight, height, smoking, use of alcohol, obesity, physical activity, family history of diabetes mellitus are all recorded. Convenient sampling was done and the sample size was calculated using the formula,

n= Z^2^ × p × (1-p) /e^2^

= (1.96)^2^ × 0.08 (1-0.08) / (0.05)^2^

= 113

Where,

n= Sample size
p= Prevalence of type 2 diabetes^5^
e= Margin of error
Z= 1.96 at 95% Confidence Interval (CI).

The calculated minimum sample size was 113, however, the total sample size taken was 114. Selection and information bias was minimized as much as possible by collecting data in the appropriately predesigned preform. Data analysis was done using Statistical Package for the Social Sciences-20 version.

## RESULTS

Out of 114 participants, the overall prevalence of diabetes mellitus was found to be 5 (4.38%). In our study, the youngest patient was 17 years of age and the eldest was 92 years of age (mean 41.05 years). The shortest was 1.42 m and the tallest patients were 1.79 m in height (mean 1.59 m). The minimum weight of the patients was 40 kg and the maximum weight of the patients was 120 kg (mean 62.39 kg). The minimum BMI of the patients was 17.3 kg/m^2^ and the maximum was 41 kg/m^2^ (mean 24.36 kg/m^2^). The minimum random blood sugar (RBS) was 69 mg/dl and the maximum was 318 mg/dl (mean 119.26 mg/dl).

Among the 5 individuals with RBS >=200, 2 (1.8%) were female and 3 (2.6%) were male in the study (Table 1).

**Table 1:** Gender wise random blood sugar.

		Female n (%)	Male n (%)	Total n (%)
**RBS**	<200	69	40	109
**(mg%)**		(60.5)	(35.1)	(95.6)
	>=200	2 (1.8)	3 (2.6)	5 (4.4)

Five (4.4%) with mild physical activities had a blood sugar level of more than 200 mg/dl while patients with 15 (13.2%) moderate or 4 (3.5%) vigorous daily activities had no one with random blood glucose level more than 200 mg/dl (Table 2).

**Table 2:** Random blood sugar and intensity of daily work.

		**RBS <200 n (%)**	**RBS >=200 n (%)**	**Total n (%)**
**Physical**	**Mild**	90	5 (4.4)	95
**activities**		(78.9)		(83.3)
	**Moderate**	15	0	15
		(13.2)		(13.2)
	**Vigorous**	4 (3.5)	0	4 (3.5)
**Total**		109	5 (4.4)	114
		(95.6)		(100)

Five (4.4%) out of 114 participants with BMI above normal had random blood sugar more than 200 mg/dl. All underweight individuals and individuals with normal BMI were found to have RBS <200 mg/dl. 46 (92%) of overweight individuals had RBS <200 mg/dl. One (50%) of the patients in the obesity class I had normal RBS and all the patients in the overweight class III group had a normal RBS, though this finding cannot accurately represent their respective group in the community since only 3 individuals were studied (Table 3).

**Table 3:** BMI classification and random blood sugar.

		**RBS <200 n (%)**	**RBS >=200 n (%)**	**Total n (%)**
**BMI Classification**	Under weight	4 (3.5)	0	4 (3.5)
	Normal	57 (50)	0	57 (50)
	Over-	46	4 (3.5)	50
	weight	(40.3)		(43.8)
	Obesity Class I	1 (0.9)	1 (0.9)	2 (1.8)
	Obesity Class II	0	0	0
	Obesity Class III	1 (0.9)	0	1 (0.9)
**Total**		109 (95.6)	5 (4.4)	114 (100)

Among the 46 participants who consumed alcohol, 5 (10.9%) people had an RBS of >=200 while 68 (100%) participants who did not consume alcohol had a normal RBS. In addition, 1 (3.85%) participants who smoked tobacco and 4. (4.54%) participants who did not smoke tobacco had an RBS of >=200 (Table 4).

**Table 4:** Random blood sugar and a history of alcohol consumption and tobacco smoking.

		**RBS <200 n (%)**	**RBS >=200 n (%)**	**Total n (%)**
**Alcohol**	Yes	41 (36)	5 (4.4)	46
				(40.4)
	No	68	0	68
		(59.6)		(59.6)
**Smoking**	Yes	25	1 (0.9)	26
		(21.9)		(22.8)
	No	84	4 (3.5)	88
		(73.7)		(77.2)

## DISCUSSION

Wild S, et al. conducted a study 2004 and concluded that the number of people with diabetes is increasing due to population growth, aging, and urbanization and increasing prevalence of obesity and physical inactivity and prevalence of diabetes are higher in men than women but there are more women with diabetes than men.^1^ Ganesh SA, et al. study showed that the prevalence of diabetes in Southern part of Chennai was 11.8% which was higher than the existing prevalence of 10.4% and concluded that more awareness creation, screening, and preventive measures need to be targeted in the population to reduce the disease burden.^2^ Singh PS et al study showed that there is a high prevalence of

type 2 diabetes in a rural area of Western Uttar Pradesh, India.^3^ Colin DM, et al. study showed that diabetes caused 1.6 million (2.8%) death in 2015, up from 1.0 million (1.8%) death in 2000.^4^ Sameer SN, et al. study also showed a high prevalence of type 2 diabetes is higher than the existing documented prevalence. Hence, more awareness creation and preventive measures need to be employed in the target population to reduce the disease burden.^6^

A study conducted in a rural area of Kouluru Village, Andra Pradesh in 2016 by Seker M, et al. showed that the awareness should be created among the rural population of Indian village and this can be done by conducting health camp and issuing the pamphlets in the rural areas where the awareness is very low.^7^ The cross-sectional study conducted in rural area of Utter Pradesh of India by Thomas T et al, in 2015 showed that mass screening for diabetes, not only provides benefits from a clinical standpoint but also helps to estimate the prevalence and hidden burden of diseases, demonstrates the exponential rise in diabetes and prompt epidemiologists to explore the related etiological mechanisms and selective screening of people aged above 40 years can have a higher of diabetic cases as evidenced in study.^8^

The study conducted in Nepal in 2015 by Gyawali B, et al. result showed that type 2 diabetes is currently a high-burden disease in Nepal, suggesting a possible area of deliberately expanding preventive interventions as well as efforts to control the disease over weight, patients were found to be more prone to develop diabetes mellitus in comparison to non-obese patients which is also indicated in the other studies. A prevalence study done in South Asia in 2012 by Jayawardena R, et al.^9^ showed that a significant epidemic of diabetes is present in the South Asia region with a rapid increase in the prevalence over the last two decades. Hence, it was suggested for an urgent need for preventive and curative strategies.^9^ A study was done in Thiruvallur district in Tamil Nadu in 2017 highlights a significant burden of undiagnosed cases of diabetes in the community and this indicates the need for systemic screening and awareness programs to identify the undiagnosed cases in the community and offer early lifestyle modification, treatment and regular follow up to such individuals.^10^ A study was done by Deepthi et al. showed that the prevalence of diabetes, IFG, and their risk factors are high among the rural population of Kolar indicating the impending diabetic epidemic in rural areas. Undiagnosed diabetes, which is a hidden danger, is high among rural areas.^11^

## CONCLUSIONS

Our result indicated that diabetes mellitus type 2 has a similar prevalence in suburban and rural areas of Nepal as some of the more developed urban areas. Since diabetes mellitus is a preventable disease if identified and treated early, regular screening of individuals with effective management at low cost can decrease the national burden and improve the quality of life. In addition, high RBS seems to be prevalent in people whose BMI falls overweight and beyond and as such should be the primary targets of regular DM screening.

## Conflicts of Interest:

**None.**
